# Re-evaluating the Role of Whole Brain Radiotherapy for Brain Metastases From Non-small Cell Lung Cancer in the Immunotherapy Era: A Retrospective Cohort Study

**DOI:** 10.7759/cureus.111399

**Published:** 2026-06-23

**Authors:** Patrick Forster

**Affiliations:** 1 Oncology, University Hospital of Wales, Cardiff University, Cardiff, GBR

**Keywords:** brain tumors (primary or brain metastases), cancer immunotherapy, neurologic complications of cancer or immunotherapy (car-t), non-small cell lung carcinoma (nsclc), sterotatic radiotherapy, whole brain radiotherapy

## Abstract

Background: The role of whole brain radiotherapy (WBRT) in non-small cell lung cancer (NSCLC) brain metastases is increasingly debated in the era of immunotherapy, with much research suggesting that its use provides little benefit for this cohort of patients.

Methods: A retrospective cohort study included patients diagnosed with NSCLC and brain metastases (2017-2020, n=147). Kaplan-Meier survival analysis and multivariate Cox proportional hazards regression were performed, and a two-tailed p-value of <0.05 was considered statistically significant.

Results: Median survival was 9.7 months, with average survival being 15.9 months from the time of diagnosis. Patients who received immunotherapy lived for 31.5 months, while those who did not lived for 14.7 months. Patients who received WBRT + immunotherapy lived an average of 19.5 months, and those who received WBRT lived an average of 10.2 months. Immunotherapy (hazard ratio (HR) 0.48, p<0.001) and stereotactic radiotherapy (SRS) (HR 0.62, p<0.001) were independently associated with improved survival. WBRT alone was associated with poorer outcomes.

Conclusion: WBRT alone provides limited benefit; immunotherapy and SRS improve survival and should be prioritized in future clinical practice.

## Introduction

Non-small cell lung cancer (NSCLC) accounts for approximately 80-85% of all lung cancer diagnoses and remains a leading cause of cancer-related mortality worldwide [1]. A significant proportion of patients with NSCLC develop metastatic disease, with the brain representing one of the most common sites. It is estimated that up to 40% of patients will develop brain metastases over the course of their illness, which are associated with a substantial increase in both morbidity and mortality [2]. The presence of intracranial disease often results in neurological deficits, reduced functional status, and a poorer overall prognosis. The management of metastatic NSCLC is complex and requires a multidisciplinary approach. Treatment decisions are influenced by several factors, including the number and location of metastases, tumor histology and molecular profile, patient performance status, and individual preferences. Current treatment modalities include systemic therapies such as chemotherapy, targeted therapy, and immunotherapy, alongside local treatments including surgery and radiotherapy. In patients with brain metastases, corticosteroids are frequently used for symptomatic relief by reducing edema and improving neurological symptoms [3].

Historically, whole brain radiotherapy (WBRT) has been a cornerstone in the management of brain metastases, particularly in patients with multiple lesions or diffuse intracranial disease. However, WBRT is associated with well-recognized adverse effects, including neurocognitive decline, fatigue, and reduced quality of life. With the emergence of more targeted treatment strategies, the role of WBRT has increasingly been questioned. In particular, the QUARTZ trial demonstrated no significant survival benefit of WBRT compared with best supportive care in selected patients with NSCLC brain metastases, raising concerns regarding its routine use [4]. Advances in systemic therapy, particularly the development of immune checkpoint inhibitors targeting pathways such as programmed death-1 (PD-1) and programmed death-ligand 1 (PD-L1), have significantly improved survival outcomes in patients with advanced NSCLC. These agents have also demonstrated intracranial activity, further challenging the traditional reliance on WBRT [5]. In parallel, stereotactic radiosurgery (SRS) has emerged as a more precise radiotherapy modality, allowing the delivery of high-dose radiation to focal lesions while sparing surrounding healthy brain tissue. SRS is associated with improved local control and a more favorable side effect profile compared to WBRT, particularly in patients with a limited number of metastases.

Increasingly, combination strategies involving systemic therapy and local treatments are being explored, with some evidence suggesting synergistic effects between immunotherapy and radiotherapy. However, outcomes remain variable, and the optimal sequence and selection of treatments are still areas of active investigation. Given the evolving therapeutic landscape, there remains uncertainty regarding the role of WBRT in the modern era. This study aims to evaluate survival outcomes in patients with NSCLC and brain metastases, with a particular focus on the impact of WBRT compared to other treatment modalities. Through survival analysis and multivariate modeling, this study seeks to identify independent predictors of outcome and to better define the role of WBRT within contemporary clinical practice.

## Materials and methods

This retrospective cohort study included patients diagnosed with NSCLC and brain metastases between January 2017 and December 2020 from across Wales. The study was conducted using routinely collected clinical data, with all data anonymized prior to analysis. To minimize potential confounding variables related to the COVID-19 pandemic, only data from the pre-pandemic period were included.

An initial database was constructed using Microsoft Excel (Microsoft® Corp., Redmond, WA, USA), comprising all patients diagnosed with lung cancer within the study period. This yielded a dataset of over 2,000 patients. Patients without a diagnosis of NSCLC were excluded, reducing the cohort to approximately 1,300 patients. From this population, patients with radiologically or histologically confirmed brain metastases were identified, resulting in a final study cohort of 149 patients.

Demographic and clinical variables collected included age at diagnosis, sex, tumor histology (adenocarcinoma versus squamous cell carcinoma), and performance status at the time of diagnosis of brain metastases. Treatment-related variables included receipt of immunotherapy, SRS, and WBRT. Given that many patients received multimodal treatment, each treatment modality was analyzed both independently and in combination where appropriate. Overall survival was defined as the time from diagnosis of brain metastases to death.

Survival analysis was performed using the Kaplan-Meier method, with survival curves compared using the log-rank test. Median and mean survival times were calculated for each treatment group. Subgroup analyses were conducted to evaluate differences in survival based on treatment modality. To identify independent predictors of survival, a multivariate analysis was performed using a Cox proportional hazards regression model. Variables entered into the model included age, sex, histology, performance status, and treatment modalities (immunotherapy, SRS, and WBRT). Hazard ratios (HRs) with corresponding 95% confidence intervals (CIs) were calculated. The proportional hazards assumption was assessed prior to model interpretation. Statistical analyses were performed using standard statistical software (e.g., SPSS). A two-tailed p-value of less than 0.05 was considered statistically significant.

## Results

**Figure 1:**
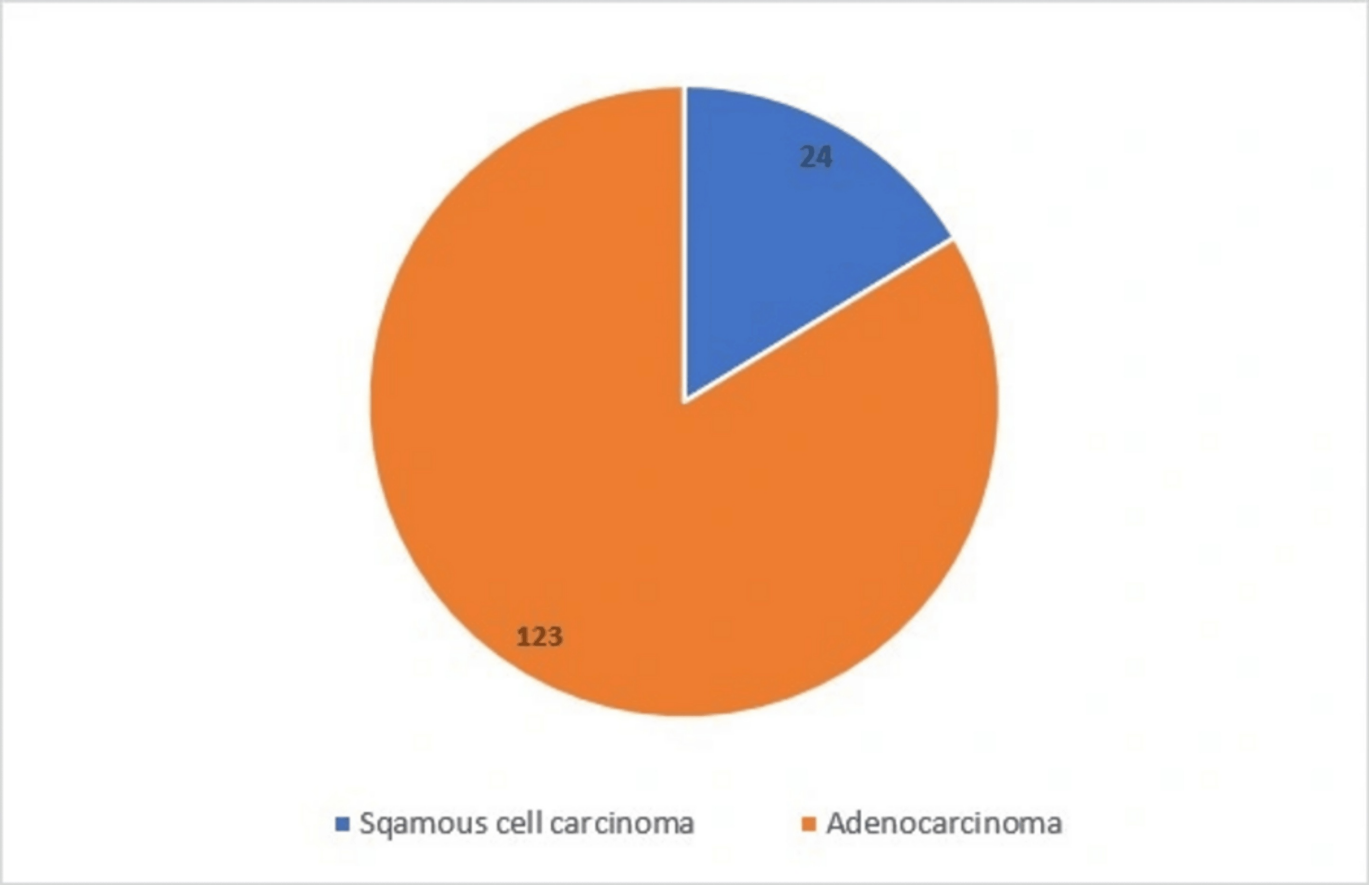
Distribution of adenocarcinoma versus squamous cell carcinoma

A total of 147 patients were included in the study, comprising 88 (60%) females and 59 (40%) males. Histological analysis demonstrated that 24 (16%) patients had squamous cell carcinoma, while the remaining 123 (84%) had adenocarcinoma (Figure 1).

**Figure 2:**
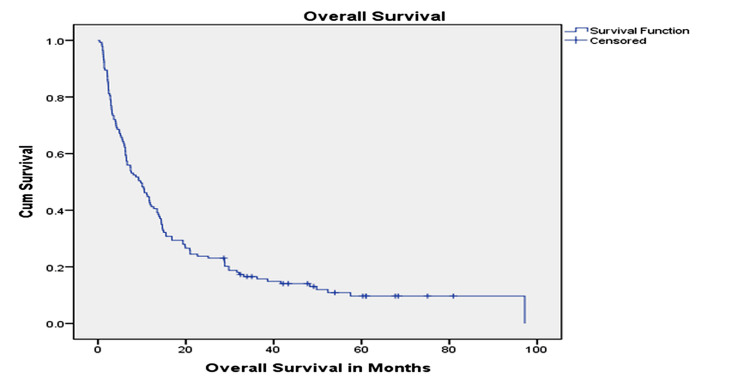
Overall survival of patients with NSCLC and brain metastases NSCLC, non-small cell lung cancer

**Figure 3:**
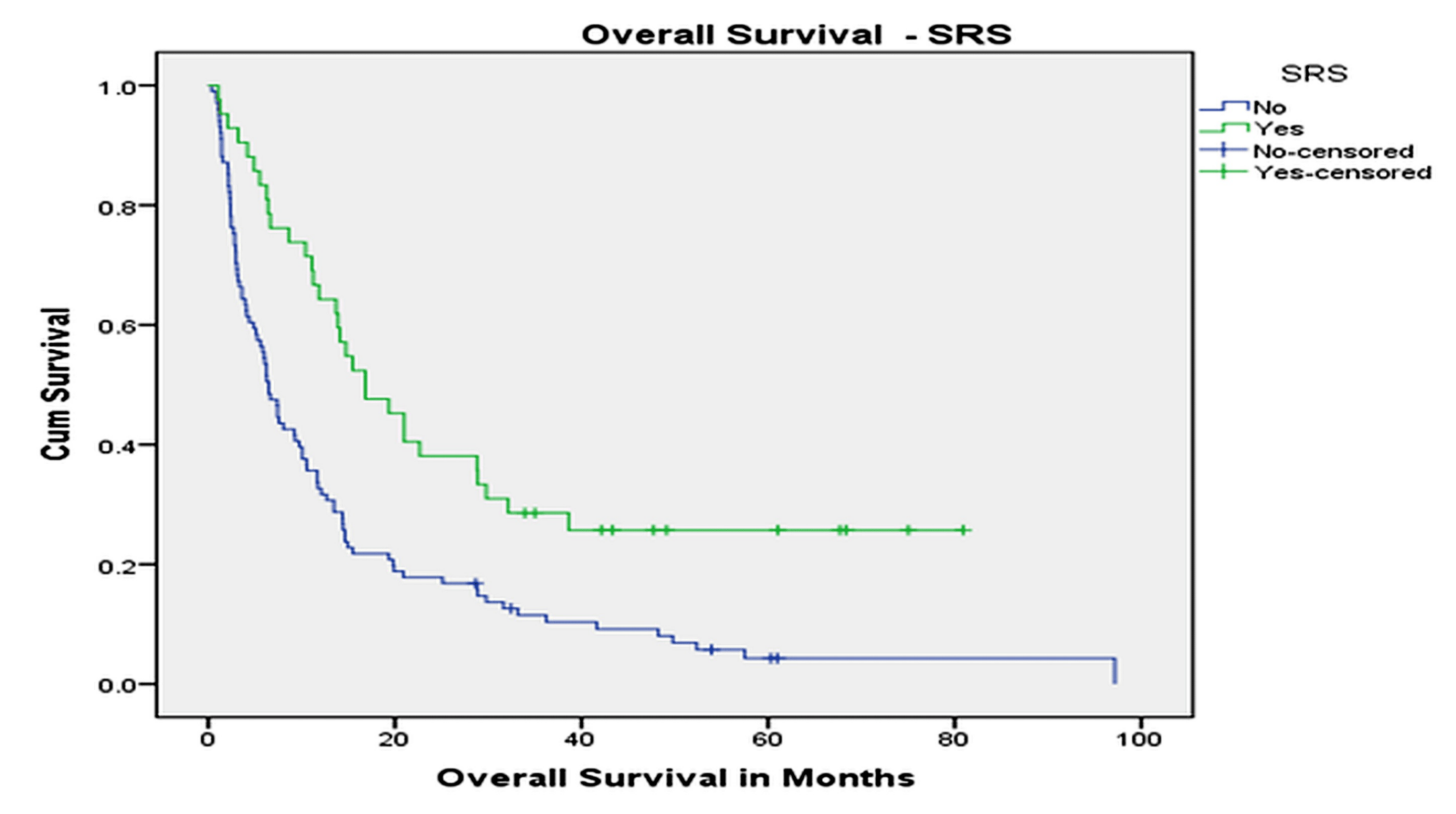
Survival in patients receiving SRS versus no SRS SRS, stereotactic radiosurgery

**Figure 4:**
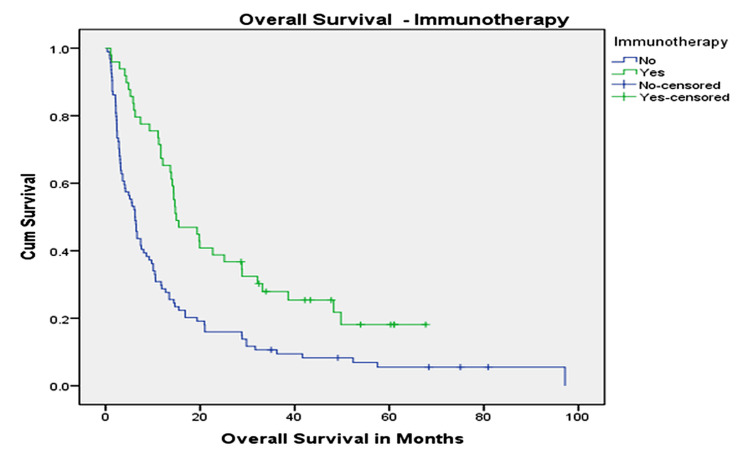
Survival in patients receiving immunotherapy versus no immunotherapy

In terms of treatment, 94 patients received immunotherapy, 59 underwent WBRT, and 42 received stereotactic radiosurgery (SRS). The majority of patients received more than one treatment modality, with 22 patients receiving a combination of WBRT and immunotherapy. The overall mean survival was 15.9 months, with a median survival of 9.7 months (Figure 2). Survival analysis demonstrated a statistically significant difference across treatment groups (p=0.005). Patients treated with SRS had a significantly longer mean survival compared to those who did not receive SRS (22.3 vs. 10.6 months, p=0.00013; Figure 3). Similarly, patients receiving immunotherapy had improved mean survival compared to those who did not (31.5 vs. 14.7 months, p<0.001; Figure 4).

**Figure 5:**
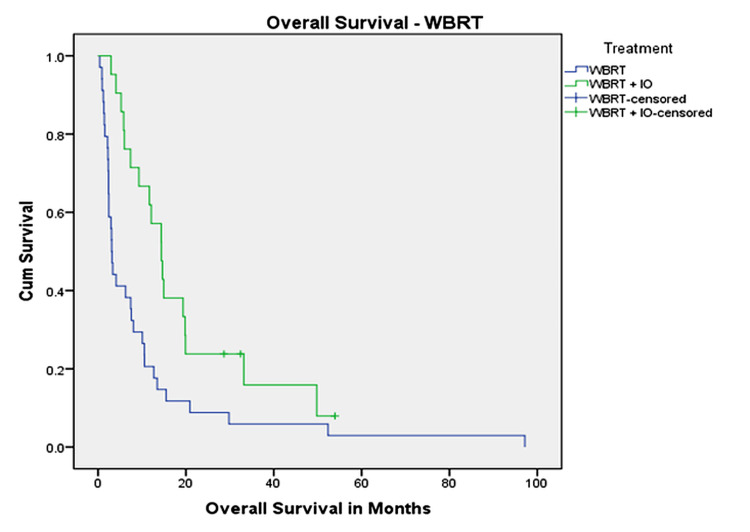
Survival in patients receiving WBRT with immunotherapy versus WBRT alone WBRT, whole brain radiotherapy; IO, immunotherapy

**Figure 6:**
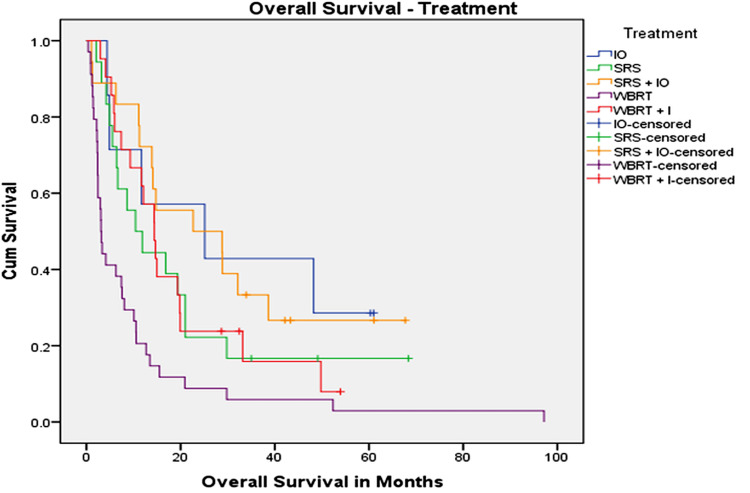
Survival by treatment modality SRS, stereotactic radiosurgery; WBRT, whole brain radiotherapy; IO/I, immunotherapy

Patients treated with a combination of WBRT and immunotherapy demonstrated improved survival compared to those receiving WBRT alone (19.5 vs. 10.2 months, p=0.001; Figure 5). Notably, survival within the first 12 months was similar across treatment groups; however, beyond 12 months, patients receiving immunotherapy alone had the longest survival, while those treated with WBRT alone had the poorest outcomes (p=0.000160; Figure 6).

Univariate analysis identified both immunotherapy and SRS as being associated with improved survival, whereas WBRT alone was associated with poorer outcomes. Specifically, immunotherapy was associated with a mean survival of 31.5 months compared to 14.7 months without immunotherapy (p<0.001), and SRS with 22.3 months compared to 10.6 months without SRS (p<0.001). WBRT alone was associated with the lowest mean survival (10.2 months). On multivariate Cox regression analysis, immunotherapy (HR 0.48, 95% CI 0.32-0.71, p<0.001) and SRS (HR 0.62, 95% CI 0.41-0.85, p<0.001) remained independently associated with improved survival. In contrast, WBRT was independently associated with worse survival outcomes (HR 1.34, 95% CI 1.05-1.72, p=0.02).

Overall, these findings demonstrate that immunotherapy and SRS are independently associated with improved survival, whereas WBRT is associated with poorer outcomes.

## Discussion

The role of WBRT in the management of metastatic NSCLC has been increasingly questioned over recent years, particularly in light of emerging systemic therapies. Historically, WBRT has been a cornerstone in the treatment of brain metastases, primarily aimed at palliation of neurological symptoms. However, contemporary evidence has challenged its survival benefit and overall clinical utility. The QUARTZ trial, a landmark randomized controlled study, demonstrated no significant improvement in overall survival or quality-adjusted life years in patients receiving WBRT compared to optimal supportive care alone. Median survival remained limited, approximately two to three months in those receiving WBRT, suggesting minimal clinical benefit in this population [6]. Furthermore, WBRT was associated with a higher incidence of adverse effects, including alopecia, fatigue, and nausea, which negatively impact patient quality of life [7]. These findings are supported by additional literature suggesting that best supportive care alone may, in selected patients, provide comparable or even superior outcomes to WBRT [8]. The evolving landscape of systemic therapies, particularly immune checkpoint inhibitors and targeted therapies, has further reduced reliance on WBRT. These modalities have demonstrated improved symptom control and survival outcomes, often with a more favorable side effect profile [9]. As such, they are increasingly considered alternatives to WBRT in the management of brain metastases from NSCLC.

Despite this, WBRT may still hold a role in carefully selected patient groups, particularly when used in combination with systemic therapies. Some evidence suggests that patients receiving WBRT alongside immunotherapy may experience improved survival compared to those receiving WBRT alone. However, this combination must be approached cautiously, as there is concern regarding increased neurotoxicity when WBRT is administered concurrently with immune checkpoint inhibitors [10]. Additionally, assessing the true survival benefit of WBRT remains challenging, as it is typically delivered with palliative intent in patients with advanced disease and poor prognosis. When comparing WBRT to SRS, current evidence suggests that SRS is associated with improved survival outcomes, with some studies reporting a mean survival advantage of over 12 months compared to WBRT. However, this comparison is confounded by selection bias, as patients receiving WBRT often have more extensive intracranial disease and poorer performance status than those selected for SRS. Consequently, the observed differences in survival may reflect underlying disease severity rather than treatment efficacy alone. Furthermore, the interpretation of survival outcomes is complicated by the multimodal nature of treatment, as many patients receive concurrent therapies such as chemotherapy, which are not always adequately accounted for in survival analyses.

Notably, patients treated with immunotherapy alone demonstrated the greatest average survival in this study. Advances in the molecular and immunohistochemical classification of NSCLC have significantly enhanced the clinical application of immunotherapy [11]. Pembrolizumab, the most commonly used agent in this cohort, is a monoclonal antibody targeting the PD-1 receptor, thereby inhibiting its interaction with PD-L1 and enhancing anti-tumor immune responses [12]. While it would be expected that higher PD-L1 expression correlates with improved response and survival, this study did not demonstrate a clear association between PD-L1 status and patient outcomes, highlighting the complexity of tumor biology and response to therapy. Emerging evidence also supports the use of immunotherapy in earlier disease stages. Neoadjuvant immunotherapy has shown promising efficacy in resectable NSCLC, with the potential to improve long-term survival outcomes [13]. This raises the possibility that immunotherapy may increasingly replace WBRT in certain clinical contexts, particularly where durable systemic and intracranial control can be achieved with fewer adverse effects. Similarly, targeted therapies have revolutionized the management of advanced NSCLC, particularly in patients with actionable mutations, leading to significant improvements in progression-free and overall survival [14,15]. In this study, patients receiving surgery or targeted therapy exhibited the longest survival, further emphasizing the importance of personalized approaches, although there was no link between PD-1 expression and survival outcomes.

However, there were several limitations to this study. The retrospective design introduces inherent biases, including variability in data collection and documentation. Data were collected by multiple clinicians, leading to potential subjectivity, particularly in the assessment of performance status, which may affect treatment decisions and survival outcomes. Additionally, while efforts were made to minimize confounding factors by using pre-pandemic data, some patients received treatment during the COVID-19 pandemic. This introduces potential bias, as healthcare delivery, treatment pathways, and patient outcomes may have been influenced by pandemic-related factors. This study demonstrates that WBRT alone is associated with inferior survival outcomes, even after adjustment for confounding variables. The multivariate analysis strengthens this finding by demonstrating that WBRT is independently associated with worse survival. Immunotherapy was the strongest predictor of improved survival, consistent with its established role in modern NSCLC management. SRS also demonstrated benefit, likely reflecting both treatment efficacy and patient selection.

## Conclusions

In this study, WBRT alone was not associated with a survival benefit in patients with NSCLC and brain metastases. While a subset of patients, particularly those with more extensive intracranial disease, may derive some benefit from combined WBRT and immunotherapy, this approach should be used cautiously given the potential for increased toxicity with concurrent treatment. SRS was associated with improved survival outcomes and represents a preferable local treatment option compared to WBRT, although its use is limited to patients with a lower burden of intracranial disease. Immunotherapy demonstrated the greatest survival benefit and remains a key component in the management of metastatic NSCLC with brain involvement. Overall, these findings suggest that optimal outcomes are achieved through a multimodal, individualized treatment strategy. The use of WBRT as a sole treatment modality should be reconsidered, as it appears to offer no survival advantage and may negatively impact quality of life compared to best supportive care. Further research could compare best supportive care with WBRT to ascertain any differences in survival outcomes between these two groups. This study supports a shift away from the routine use of WBRT in favor of more targeted approaches. Personalized treatment strategies incorporating immunotherapy and SRS, where appropriate, should be prioritized, as both were independently associated with improved survival outcomes.

## References

[1] Patil SM, Kunda NK (2022). Anticancer activity of D-LAK-120A, an antimicrobial peptide, in non-small cell lung cancer (NSCLC). Biochimie.

[2] Buriolla S, Pelizzari G, Corvaja C (2022). Immunotherapy in NSCLC patients with brain metastases. Int J Mol Sci.

[3] Gaebe K, Li AY, Park A (2022). Stereotactic radiosurgery versus whole brain radiotherapy in patients with intracranial metastatic disease and small-cell lung cancer: a systematic review and meta-analysis. Lancet Oncol.

[4] Mulvenna P, Nankivell M, Barton R (2016). Dexamethasone and supportive care with or without whole brain radiotherapy in treating patients with non-small cell lung cancer with brain metastases unsuitable for resection or stereotactic radiotherapy (QUARTZ): results from a phase 3, non-inferiority, randomised trial. Lancet.

[5] Li Z, Zhang S, Wang Y, Yan Y (2025). The role of exosomal PD-L1 in NSCLC immunotherapy. Immunotherapy.

[6] Jones JA, Simone CB 2nd (2015). Whole brain radiotherapy for patients with poor prognosis: possibilities for the impact of the QUARTZ trial. Ann Palliat Med.

[7] Bruynzeel AM, Lagerwaard FJ (2016). Whole brain radiotherapy for brain metastases from non-small cell lung cancer: the end of an era?. J Thorac Dis.

[8] Nieder C, Norum J, Dalhaug A, Aandahl G, Pawinski A (2013). Radiotherapy versus best supportive care in patients with brain metastases and adverse prognostic factors. Clin Exp Metastasis.

[9] Bui N, Woodward B, Johnson A, Husain H (2016). Novel treatment strategies for brain metastases in non-small-cell lung cancer. Curr Treat Options Oncol.

[10] Bjørnhart B, Hansen KH, Asmussen JT, Jørgensen TL, Herrstedt J, Schytte T (2022). Effect and tolerability of immunotherapy in patients with NSCLC with or without brain metastasis. Cancers.

[11] Osmani L, Askin F, Gabrielson E, Li QK (2018). Current WHO guidelines and the critical role of immunohistochemical markers in the subclassification of non-small cell lung carcinoma (NSCLC): moving from targeted therapy to immunotherapy. Semin Cancer Biol.

[12] Francisco LM, Sage PT, Sharpe AH (2010). The PD-1 pathway in tolerance and autoimmunity. Immunol Rev.

[13] Uprety D, Mandrekar SJ, Wigle D, Roden AC, Adjei AA (2020). Neoadjuvant immunotherapy for NSCLC: current concepts and future approaches. J Thorac Oncol.

[14] Jackson CM, Lim M, Drake CG (2014). Immunotherapy for brain cancer: recent progress and future promise. Clin Cancer Res.

[15] Zhou K, Cai X, Wang X, Lan X, Zhang X (2022). Efficacy and safety of WBRT+EGFR-TKI versus WBRT only in the treatment of NSCLC patients with brain metastasis: an updated meta-analysis. Thorac Cancer.

